# Research on the impact mechanism of comprehensive land consolidation on rural human settlement environment based on the production-living-ecological space theory: a case study of Zhejiang Province, China

**DOI:** 10.3389/fpubh.2025.1633895

**Published:** 2025-09-11

**Authors:** Lin Meng, Fengjuan Yan

**Affiliations:** School of Public Administration, Shandong Normal University, Jinan, China

**Keywords:** comprehensive land consolidation, rural human settlement environment, production-living-ecological space theory, influence mechanism, Zhejiang Province, China

## Abstract

Exploring the influence mechanism of Comprehensive Land Consolidation (CLC) on the quality of Rural Human Settlement Environments (RHSE) is essential for advancing theoretical and methodological advancements in RHSE improvement. Additionally, this study provides a reference for implementing China’s rural revitalization strategy and advancing CLC pilot programs. In this article, Zhejiang Province was selected as the research area, where entropy methods, geographic detectors, and other analytical tools were employed to examine the impact mechanisms of CLC on RHSE. Policy recommendations were proposed based on the findings. The results showed that: (1) At both the provincial and municipal levels, both RHSE and CLC exhibited significant improvements in Zhejiang from 2000 to 2020, confirming the pivotal role of CLC in enhancing RHSE quality. (2) The single factor detection results of the geographic detector indicated that CLC has high explanatory power for improving RHSE quality by promoting an increases in average grain yield per mu, public land area, land use elasticity, effective irrigation rate, concentrated and contiguous farmland, land reclamation rate, and other factors. The results revealed the important role of CLC in improving the average grain yield per mu, effective irrigation rate, concentrated contiguous farmland, and land reclamation rate in driving RHSE development. (3) Based on the research results, various CLC policies can be implemented to improve the RHSE. The government can integrate high-standard farmland construction to enhance the potential for agricultural development. Meanwhile, policies can be formulated to promote industrial chain extension and advance infrastructure construction. Additionally, policies can be proposed to raise awareness of vegetation protection and improve ecological restoration work

## 1 Introduction

Rural Human Settlement Environment (RHSE) palys a crucial role in farmers’ livelihoods, sustainable rural development, and national stability (1). In recent years, despite China’s rapid economic growth, challenges including fragmented land use, environmental degradation, and inefficient resource allocation have persisted in rural areas (2). The extensive and unregulated development model has resulted in increasingly prominent issues including disorderly spatial layout of agriculture and rural areas, inefficient resource utilization, deteriorating ecological quality, abandoned land, and fragmented farmland. Along with these issues come a series of environmental pollution problems (3).

For a long time, land consolidation has played a significant role in addressing rural land use issues during development, promoting urban–rural integration, and building beautiful rural areas (4). Firstly, Comprehensive Land Consolidation (CLC) has emerged as a strategic tool for promoting the improvement of RHSE quality by optimizing spatial layouts, enhancing land productivity, and promoting ecological restoration (5). The Ministry of Natural Resources issued a notice on carrying out pilot work for CLC across the country at the end of 2019, proposing to uniformly deploy and carry out pilot work for CLC across the country. CLC emphasizes multiple dimensions, including “connotation, objectives, means, and benefits.” CLC is an effective measure for optimizing rural spatial layouts, promoting rural spatial reconstruction, and addressing the issue of rural land resource allocation (6, 7). Secondly, as a key measure to protect arable land and permanent basic farmland, enhance the level of land conservation and intensive use, and strengthen ecological restoration platforms, CLC is an important strategic deployment for implementing the rural revitalization strategy (8), which helps to improve the quality of RHSE (1, 4, 7). As a means of coordinating the relationship between people and land, CLC is achieved through resource integration and element cohesion, adhering to the three priority principles of “ecology, protection, and conservation” (5). CLC can promote the ecological living of rural population, the ecological management of rural land, and the ecological transformation of rural industries, thereby achieving high-level construction of RHSE (1, 6). Therefore, it is of great research significance to study the influence mechanism of CLC on the quality of RHSE, and formulate policies to improve the quality of RHSE based on this.

The production-living-ecological space theory posits that, from the perspective of land use functions, rural space can be categorized into production space, living space, and ecological space (6). Both the comprehensive improvement of land across the entire region and the rural human settlement environment exist within the rural space, and are closely associated with the production, living, and ecological spaces. Some studies have initially verified that the CLC exerts an impact on the RHSE by influencing the rural production, living, and ecological spaces. Firstly, in terms of elements, CLC reflects the intrinsic relationship between the rural population, land, and industry. In terms of functionality, CLC reflects the practical needs of farmers for production, life, and ecology. In terms of structure, CLC presents multidimensional characteristics of element quantity, quality, and space (4, 9, 10). Secondly, CLC can effectively agglomerate factors such as population, land, and industries, optimize the quantity, quality, and spatial structure of different essential factors, enhance the production space, and provide a driving force for realizing the optimization of RHSE quality (6). Thirdly, taking respect for farmers’ will as the prerequisite, the CLC rationally conducts rural settlement planning and the renovation of “hollow villages” (1) It guides the orderly withdrawal of idle rural construction land, coordinates various types of land use such as rural housing construction and public services, optimizes living space, and promotes the orderly and appropriate concentrated residence of the rural population (7, 11, 12). Fourthly, through measures such as land reclamation and ecological restoration, the CLC is equipped with modern residential environments and complete living facilities. From the perspective of optimizing ecological space, it has effectively improved the quality of t RHSE (5). However, existing studies have rarely systematically analyzed the impact of the comprehensive improvement of land across the entire region on the human settlement environment (5, 13).

**Figure 1:**
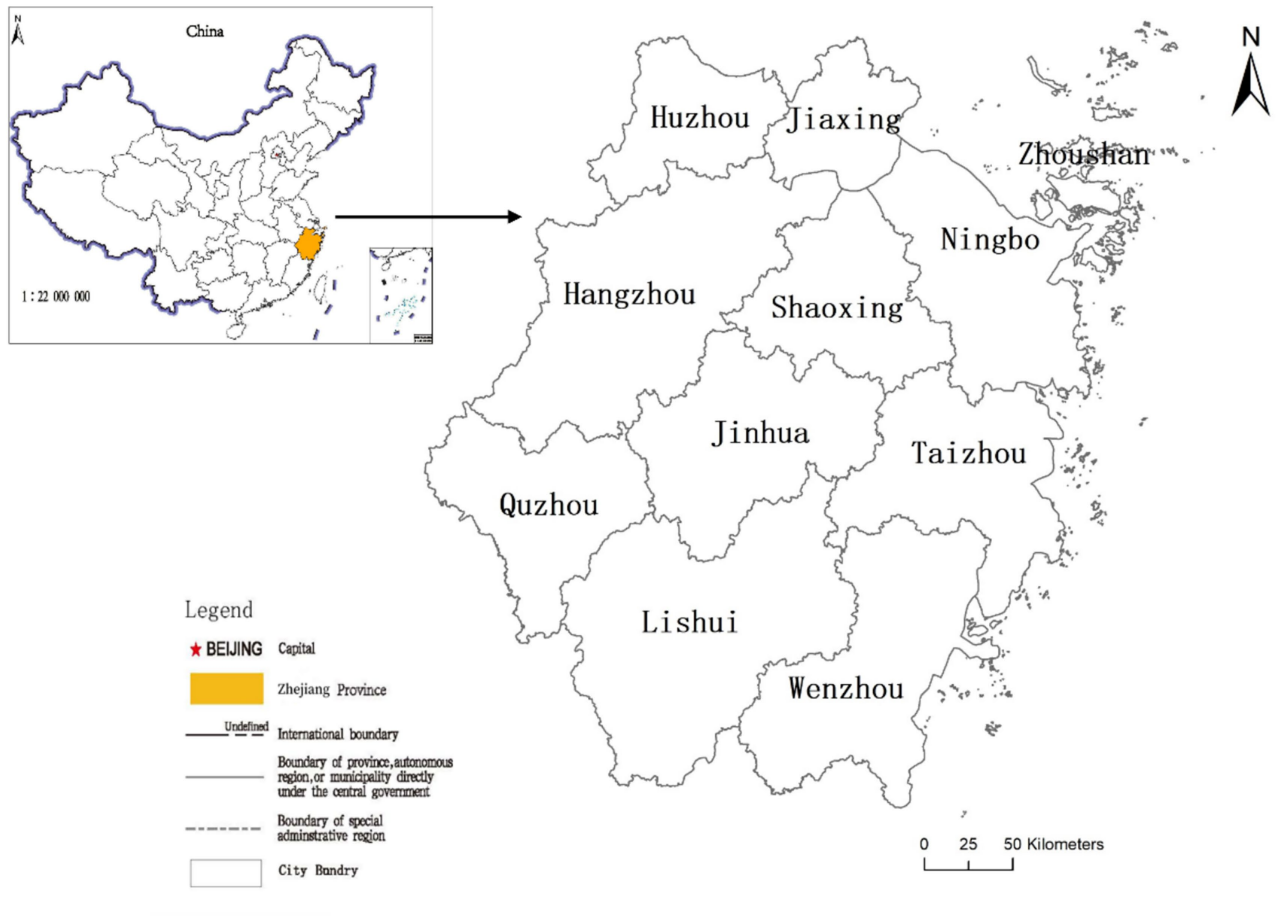
The location of the study area in China.

Zhejiang Province is situated on the southeast coast of China, featuring a subtropical monsoon climate with distinct monsoons and four distinct seasons (Figure 1). Over the past 20 years, the Green Rural Revival Program has been consistently and comprehensively implemented in Zhejiang Province through the utilization of CLC. Through CLC, the integration of urban and rural development has been further advanced, rural industries have thrived, and the transformation of RHSE has been realized. Work related to agriculture, rural areas, and farmers has achieved historic, pioneering, and leading accomplishments in Zhejiang Province. In September 2018, the Green Rural Revival Program in Zhejiang Province was awarded the highest environmental honor by the United Nations Environment Programme—the Champions of the Earth Award.

From 2000 to 2020, rural socio-economic development in Zhejiang Province exhibited diverse characteristics (Table 1). Through village consolidation and spatial layout optimization in the context of CLC, the number of village committees continued to decrease, dropping from 42,226 to 19,806. Benefiting from the improved efficiency of large-scale land management following CLC, the added value of the primary industry showed an overall growth trend, fluctuating from 630.97 billion yuan to 2,166 billion yuan. With population agglomeration toward towns or central villages and the optimization of the urban–rural population structure, the rural population in Zhejiang Province showed a fluctuating downward trend. However, the integration and efficient utilization of land resources through CLC have stabilized the areas of agricultural land and cultivated land (It should be noted that due to the adjustment of statistical standards for cultivated land in China’s land use change survey in 2019, a significant decrease was observed in cultivated land statistical data). Overall, CLC has provided crucial support for the adjustment of rural economic structures and the transformation of social patterns.

**Table 1:** Main rural socio-economic indicators of Zhejiang Province.

Indicators year	Number of village committees (unit)	Added value of primary industry (100 million yuan)	Rural population (10,000 people)	Agricultural land (10,000 hectares)	Cultivated land (10,000 hectares)
2000	42226.00	630.97	2357.40	872.96	208.93
2001	40569.00	659.78	3631.31	871.45	208.17
2002	39125.00	685.20	3664.10	868.99	204.61
2003	38322.00	717.85	3712.12	866.28	203.04
2004	35445.00	803.83	3734.83	864.08	199.86
2005	34515.00	881.47	3790.50	871.15	194.77
2006	32976.00	913.16	3770.17	872.66	191.65
2007	31060.00	969.27	3770.46	869.88	191.75
2008	30068.00	1073.30	3761.72	867.15	192.09
2009	29958.00	1134.68	3778.86	871.16	198.67
2010	29303.00	1322.85	3813.24	869.26	198.37
2011	28812.00	1535.20	3845.61	867.59	198.16
2012	28798.00	1610.81	2016.00	866.07	197.94
2013	28339.00	1718.74	3993.86	864.21	197.85
2014	27997.00	1726.57	1935.00	862.43	197.66
2015	27901.00	660.65	1894.30	861.22	197.79
2016	27568.00	1890.40	1844.70	859.88	197.46
2017	27458.00	1934.00	1810.20	858.89	197.70
2018	24711.00	1976.00	1784.20	858.02	197.93
2019	20402.00	2087.00	1755.00	869.54	129.05
2020	19806.00	2166.00	1800.04	868.03	128.10

The existing achievements have provided a lot of theoretical guidance and methodological inspiration for CLC and improvement the quality of RHSE. However, research on the impact of CLC on RHSE quality remains insufficient. Due to the late initiation of research on CLC, existing studies primarily consider CLC as a macro background and evaluate RHSE quality in specific areas. There is little literature that directly studies the impact of CLC on RHSE quality.

Therefore, this article takes Zhejiang Province, China as the research area, cities as the research unit, and the period from 2000 to 2020 as the research period to explore the influence mechanism of CLC on RHSE. Firstly, a theoretical analysis framework for the impact of CLC on RHSE was established. Secondly, the CLC level indicator system and RHSE quality indicator system were developed. Secondly, CLC level indicator system and RHSE quality indicator system were established. The entropy method was used to calculate the weights of the indicators, and to obtain the evolution of CLC levels and RHSE quality in the study area for the years 2000, 2005, 2010, 2015, and 2020. Meanwhile, the geographic detector model was used to reveal the inherent mechanism of CLC, which effectively drives the improvement of RHSE through single-factor and multi-factor interactions. Finally, based on the calculation results, a differentiated policy for CLC-driven optimization of RHSE quality was formulated to promote the improvement of RHSE quality.

In terms of research innovation, this study makes two key contributions. First, based on the production-living-ecological space theory, it systematically sorts out the theoretical influence mechanism of CLC on RHSE. Second, it further quantitatively verifies the impact of CLC on RHSE by applying the Geodetector method.

Regarding the research significance, the findings of this study contribute to two key aspects. On one hand, they facilitate the improvement of theories and methods for enhancing the quality of RHSE. On the other hand, by integrating existing experience and developing differentiated strategies for optimizing the RHSE, they can provide references for the implementation of rural revitalization strategies and the advancement of CLC pilot projects.

## 2 Theoretical analysis framework for the influence mechanism of CLC on RHSE

### 2.1 Definition of CLC

CLC represents a further evolution of land consolidation (14). In 2003, with CLC as the starting point, the Green Rural Revival Program was launched in Zhejiang Province. The 2019 Notice of the Ministry of Natural Resources on Conducting Pilot Work on CLC Nationwide proposed that CLC is a comprehensive management activity based on scientific and rational planning, with townships as the basic implementation units (the consolidation area may cover all or part of the villages within a township). Through the comprehensive promotion of agricultural land consolidation, construction land consolidation, and rural ecological protection and restoration, CLC optimizes the spatial pattern of production, living, and ecology, and implements comprehensive management of national land space in areas that are idle, underutilized, ecologically degraded, or environmentally damaged. Thus, CLC can promote the protection of arable land, the intensive and economical use of land, the improvement of RHSE, and the comprehensive revitalization of rural areas (7).

Compared with traditional land consolidation, CLC adopts a holistic perspective. CLC emphasizes the integrity of the consolidation scope, the comprehensiveness of the consolidation objects, the diversity of consolidation modes, the systematicness of consolidation measures, and the multiplicity of consolidation goals. CLC focuses on strengthening the supportive capacity of land use for rural development (9), achieving a supply–demand balance between land use supply and rural development demand (10). The ability of the rural land use system to withstand external shocks is enhanced, facilitating the sustainable development of national land space (4, 6).

Supported by land engineering, infrastructure engineering, environmental engineering, and ecological engineering, comprehensive and systematic governance of rural mountains, waters, forests, fields, lakes, and grasslands is achieved through the comprehensive promotion of agricultural land consolidation, construction land consolidation, and rural ecological restoration efforts under CLC. Rural society, economy, and ecological space are reconstructed, while production functions, living conditions, and the ecological environment in rural areas are improved (7, 11).

### 2.2 Definition of RHSE

Geddes’ (45) “Human Settlement” and Wu et al. “Sciences of Human Settlements” are two major milestones in the systematic study of human settlement science in both China and the West. Urban planning scholars from developed Western countries proposed the theory of rural cities and regional concepts as early as the early 20th century (15–17). Later, Doxiadis proposed the establishment of a discipline of human settlement studies to guide urban and rural development (18). In China, Wu argues that the human settlement system should include five systems as follows: natural system, human system, social system, residential system, and supportive system (19). In the 21st century, against the backdrop of new rural construction, research on RHSE in China is steadily increasing.

RHSE refers to the place where humans reside and live, and it is the surface space closely related to human survival activities in rural areas. The core of RHSE is people, and the purpose of its construction is to meet the needs of human settlement (2). Based on Mr. Wu’s theory, RHSE can be divided into five subsystems: natural, human, residential, social, and supportive systems. Among them, the natural system is mainly composed of natural environmental elements such as hydrology, meteorology, and topography. The human system mainly involves human material and spiritual needs, etc. The social system mainly includes human communication systems such as education and healthcare. The residential system mainly refers to the spatial carrier that provides shelter for human activities. The supportive system mainly involves the infrastructure of human living and activity places. These five systems establish the content dimensions of the human settlement environment and constitute the basic elements of the human settlement environment system (19–21).

### 2.3 Theoretical analysis framework for the impact of CLC on RHSE

**Figure 2:**
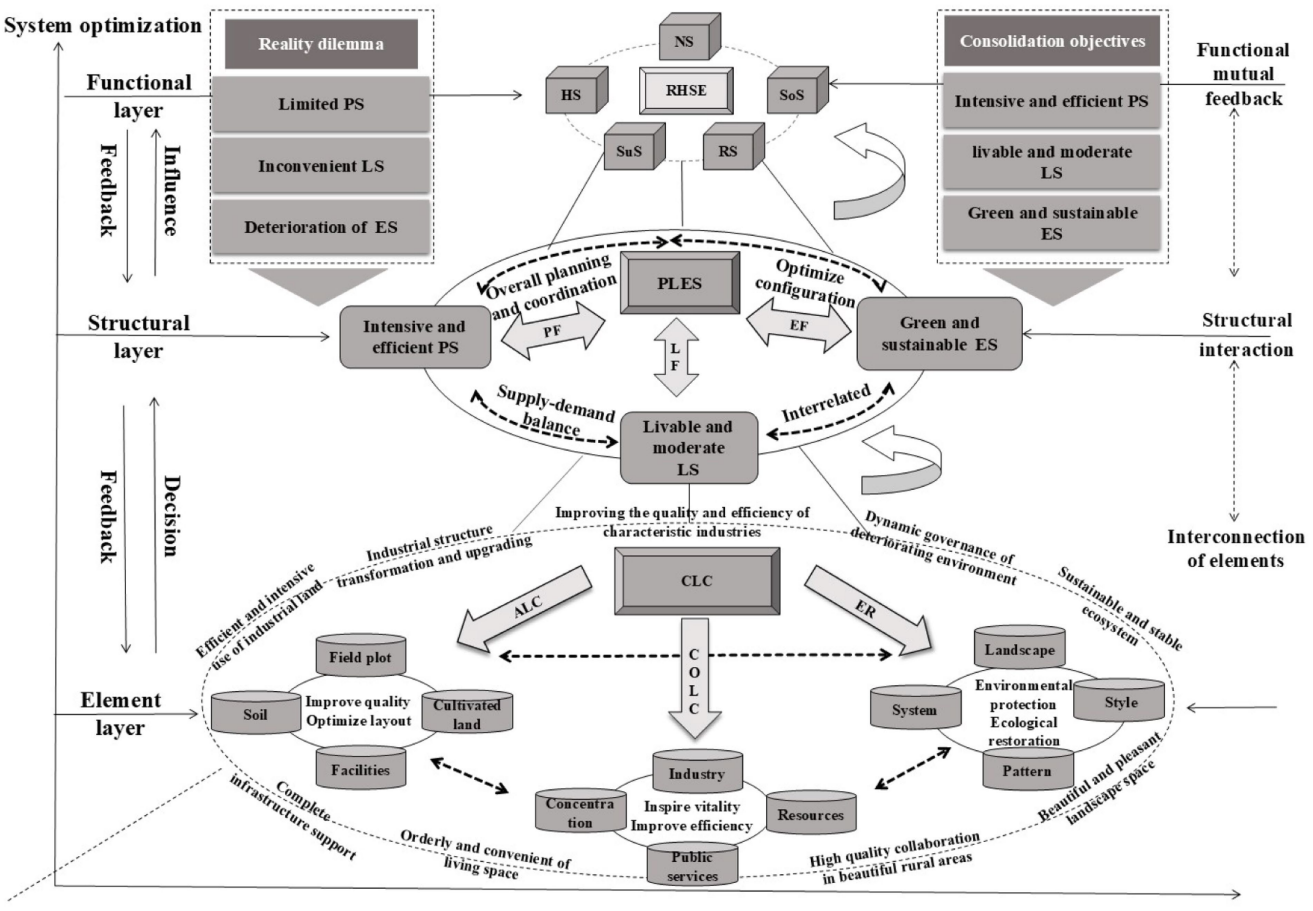
The relationship between CLC and RHSE. ALC represents agricultural land consolidation; CoLC represents construction land consolidation; ER represents ecological restoration; CLC represents comprehensive land consolidation; PLES represents production-living-ecological space; RHSE represents rural human settlement environment; PS represents production space; LS represents living space; ES represents ecological space; NS represents natural system; HS represents human system; SoS represents social system; RS represents residential system; SuS represents supportive system; PF represents Production Function; LF represents Living Function; EF represents Ecological Function.

CLC adjusts factors such as land, population, and industry in rural areas, alters the production-living-ecological spatial structure, and optimizes the functions of RHSE (6). From the perspective of systems theory, a system exists in three dimensions: elements, structure, and function (22–24). The combination of elements within the system determines its structural form, and changes in structure will affect the system’s overall functionality (25). Firstly, in China’s rural system, elements refer to factors such as land, population, industry, and ownership that are influenced by CLC. They serve as the foundation and carrier of CLC (4, 26). Secondly, structure refers to the combination of elements. Production-Living-Ecological Space is proposed by China from the perspective of land use function during the implementation of rural revitalization policies, including production space, living space, and ecological space (26–28). Finally, function refers to the manifestation of the CLC effect across the entire area, reflected as the function of providing human welfare (29). In other words, it refers to the function of improving the rural living environment (Figure 2).

**Figure 3:**
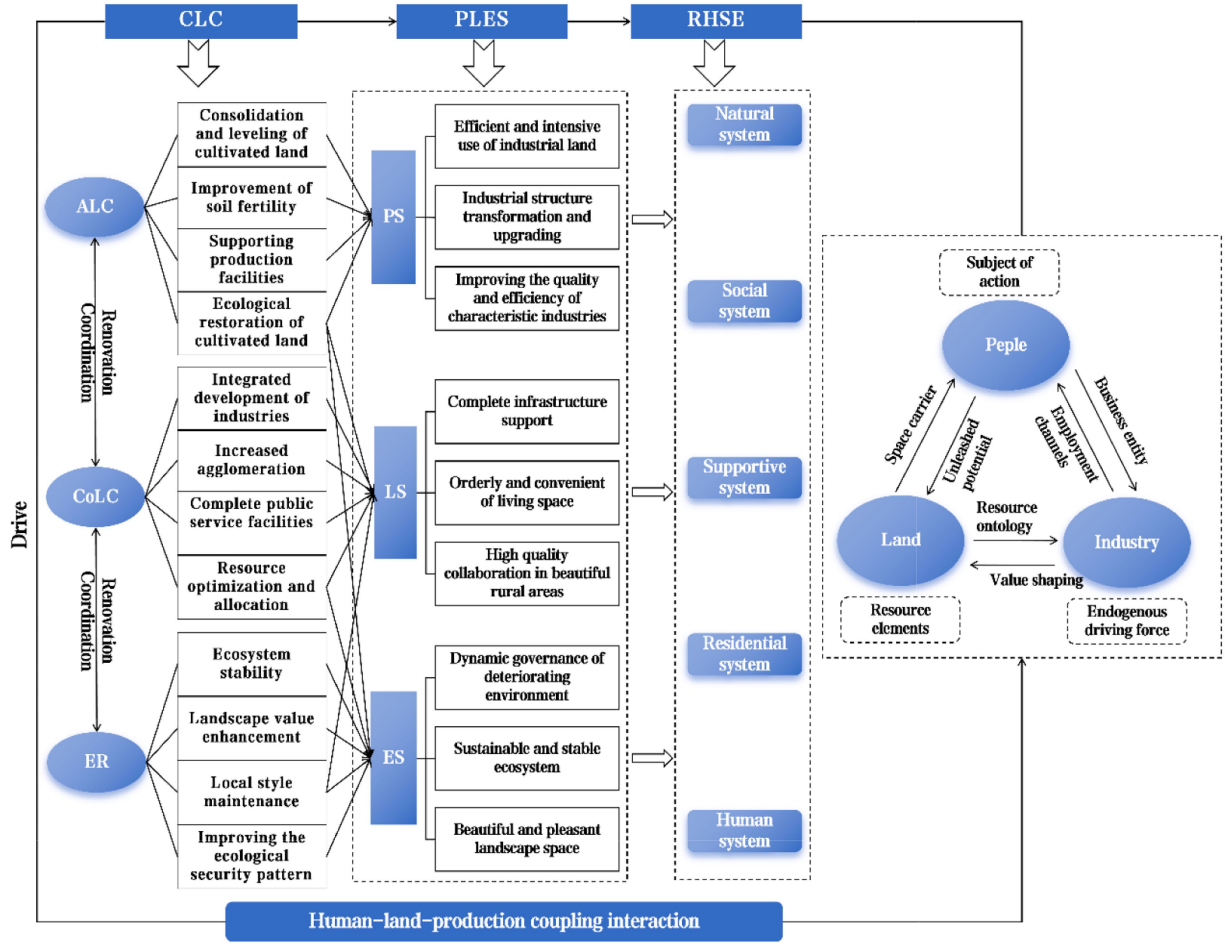
Theoretical analysis framework for the impact of CLC on RHSE guided by the “Production-Living-Ecological Space “function. ALC represents agricultural land consolidation; CoLC represents construction land consolidation; ER represents ecological restoration; CLC represents comprehensive land consolidation; PLES represents production-living-ecological space; RHSE represents rural human settlement environment; PS represents production space; LS represents living space; ES represents ecological space.

Firstly, CLC is based on scientific planning, involving the promotion of agricultural and construction land consolidation, as well as rural ecological protection and restoration. The relationships between factors such as land, population, and industry is improved (6). Secondly, the adjustment of elements has optimized the spatial pattern of production, living, and ecology within villages, achieving a structural adjustment of rural production-living-ecological space (26). Finally, the optimization effect of production-living-ecological space on the rural regional system brings about the functional optimization and value diffusion of the system, thereby improving RHSE quality (27) (Figure 3). Specifically, CLC improves the RHSE quality through the following aspects.

#### 2.3.1 CLC improves the quality of RHSE by promoting intensive and efficient of production space

The development of rural industries is fundamental to rural construction. As a value-added carrier of production space, rural land provides resources and space for industrial development (5). The key factors limiting the production space of RHSE are the fragmentation of rural farmland, extensive land use, scattered and inefficient industrial land, and disorderly and lagging industrial development. CLC promotes the agglomeration of production space through the restructuring of agricultural and non-agricultural production parcels (26). The layout of rural production space is optimized and the structure is adjusted (6, 26), meeting human demand for material products and improving the living environment in rural areas (27).

##### 2.3.1.1 CLC improves the quality of RHSE by enhancing agricultural production functions of production space

In terms of agricultural land consolidation, CLC intervenes in rural agricultural production behavior and spatial layout through agricultural land consolidation engineering techniques such as high standard farmland construction, inefficient forest land, grassland, and garden land consolidation, farmland quality improvement and transformation, and farmland infrastructure construction (7). CLC solves the problem of fragmented spatial layout by constructing a modern agricultural new pattern of fields forming squares, canals forming networks, roads connecting, and ditches interlinking (30). Firstly, arable land is the foundation of the livelihood of rural residents and the basic source of their sustenance (11). Combining agricultural land consolidation with high standard basic farmland construction (31), to construct high-standard basic farmland and increase the area of effective cultivated land, agricultural land consolidation implements land leveling projects featuring “merging small plots into large ones” and corresponding land ownership adjustment measures. These efforts enable the contiguous development and re-layout of fragmented and scattered cultivated land, which increases the proportion of organic matter in the topsoil of cultivated land and the thickness of effective soil layers. Consequently, this improves land quality, enhances soil fertility, promotes the ecological restoration of rural cultivated land, and optimizes the natural system of RHSE (1, 7). Secondly, to increase the average grain yield per mu, through agricultural water conservancy projects and field road engineering, agricultural land consolidation is equipped with corresponding irrigation and drainage facilities and improves field and production roads. The farmland infrastructure is optimized to facilitate agricultural irrigation and drainage, as well as farmers’ production and transportation (5). These measures effectively promote large-scale mechanized agricultural development, and improve the supportive system of RHSE. Thirdly, to increase per capita net income of villagers and the net output value of grain per mu, through modern facilities, agricultural land consolidation improves agricultural production efficiency, enhanced land output benefits, and promoted the improvement and efficiency of rural production space (4, 9). The production of agricultural products and food security in rural production spaces are strengthened (11), improving the human system of RHSE. These measures not only meet the livelihood needs of farmers, but also increase the effective supply of grain production, creating conditions for moderate scale operation of agriculture and the development of modern agriculture.

Construction land consolidation improves the human system and supportive system of RHSE by enhancing agricultural production functions. Firstly, to increase net output value of grain per mu, through the consolidation of idle and inefficient construction land, construction land consolidation can relocate scattered farmhouses in farmland and reclaim abandoned homesteads (30). Construction land consolidation increases the area of agricultural land effectively, reduces the level of fragmentation of arable land, and concentrates farmland in contiguous areas (4, 14). Agricultural production is more convenient and efficient, crop production is scaled up, and the human system of RHSE is enhanced. Secondly, in the process of constructing high standard farmland and upgrading cultivated land, rural road networks, agricultural water conservancy facilities, energy and electricity infrastructure will be improved (5). The perfect facilities lay the foundation for the scale and mechanization of agricultural operations (6), Improving the supportive system for RHSE.

Ecological restoration improves the human and natural systems of RHSE by enhancing agricultural production functions. Firstly, to increase average grain yield per mu, through land leveling and consolidation, remediation of mining-affected land and soil, and control of soil erosion, the soil structure is improved (32). At the same time, the soil quality, fertility, and water retention capacity are enhanced (33). The improvement of environmental conditions for crop growth has led to an increase in grain yield (9), and improved the human system of RHSE. Secondly, Through the preservation of traditional cultivation and management techniques such as straw returning to the field and composting in the construction of high-standard basic farmland, the conversion of organic matter in the soil is promoted, the absorption of available soil nitrogen by crops is increased, and nutrient balance is maintained, thereby improving the rural ecological environment and enhancing the quality of the natural system (10).

##### 2.3.1.2 CLC improves the quality of RHSE by improving non-agricultural production functions of production space

Agricultural land consolidation enhances the human system and supportive system of RHSE by improving non-agricultural production functions. Firstly, to increase average grain yield per mu, the consolidated farmland is centrally transferred to large-scale grain growers or other new business entities for modern agricultural production (34). The concentration of farmland can not only improve agricultural production efficiency, but also increase employment opportunities for farmers (31). The production and living conditions of farmers are improved (4), and their livelihood needs are met (10), optimizing the human system of RHSE. Secondly, after the construction of high-standard farmland, in the process of comprehensive land consolidation, the degree of fragmentation of agricultural land has been reduced, and irrigation and drainage facilities and rural road networks have been improved (7). On the one hand, it has driven the construction of rural infrastructure and improved the supportive system of RHSE. On the other hand, new business entities are attracted to engage in agriculture, accelerating the concentration and accumulation of capital and technology (34), thereby creating a favorable investment environment for local business entities and foreign investors (1), promoting the exchange of resources such as funds and technology (6). These measures have driven the rise of agricultural product processing industry and leisure tourism industry. The production and development potential of agricultural land was released, and the value of rural land use was enhanced (11), while the human system of RHSE is improved.

Construction land consolidation improves the living system, human system, and social system of RHSE by improving non-agricultural production functions. Firstly, to increase per capita net income of villagers, the consolidation of existing construction land can revitalize inefficient rural construction land. Rural construction land is orderly vacated, leaving space for moderate agglomeration of rural industrial and commercial development and optimizing the spatial layout of non-agricultural production in rural areas (6). On the premise of complying with village planning, construction land consolidation is carried out through the revitalization or voluntary withdrawal of construction land such as idle rural homesteads and public facilities land, and reclaiming them for a fee. After land consolidation, they are put on the market as collectively operated construction land (11). The entry of collectively operated construction land into the market has expanded the space for construction and development, addressed the issue of scattered and inefficient rural land layout, enhancing the living system of RHSE. Secondly, the consolidation of construction land provides usable land for rural industrial and commercial development, enabling the reuse of previously idle and abandoned industrial and residential land (6). At the same time, measures such as linking increase and decrease, balancing the occupation and compensation of arable land, and introducing social capital are taken to replace land indicators or increase external investment. The transaction value, production value, and management value of land are activated, and social capital and new business entities enter rural areas (7). Moreover, through the introduction of advanced technology, information, and knowledge as production factors into rural areas, combined with the balanced allocation and orderly integration of various factors, modern industries and specialized agriculture are developed in a chain like manner, releasing the potential for rural development and promoting the integration of new industries and formats in rural areas (31). These measures have enriched rural industrial formats and provided more employment opportunities for rural residents. The social employment function of land is demonstrated, improving the human system of RHSE. Thirdly, the rural tourism, agricultural sightseeing and other service industries formed through the consolidation of construction land have attracted a large number of villagers to participate, which is not only beneficial for increasing farmers’ income and improving agricultural efficiency, but also can promote the deep integration of agriculture and tourism (11). The function of rural land in ensuring social livelihood is realized, meeting the diverse needs of villagers and tourists, and optimizing the social system of RHSE.

Ecological restoration enhances the human and residential systems of RHSE by improving non-agricultural production functions. Firstly, to support the construction of high-standard farmland and increase grain yield, by implementing ecological restoration projects such as farmland ecosystem protection, biodiversity maintenance, and ecological landscape pattern construction, the ecological environment of the village is optimized. At the same time, relying on the natural and beautiful rural scenery, comfortable and pleasant fresh climate, and environmentally friendly green spaces, green, low-carbon, and healthy tourism venues such as garden hotels and health centers are being built in rural areas. The development of rural ecological tourism model aims to improve the quality and added value of agricultural products to increase farmers’ income, and promote green and sustainable development of agricultural land, improving the human and natural systems of RHSE. Secondly, in the process of ecological protection and restoration, natural landscapes and traditional cultural resources are excavated, promoting a positive interaction between industrial development and the protection and revival of characteristic cultures (35). The production and lifestyle in rural areas are changed, creating conditions for creating a new pattern of ecological livability, intensive and efficient rural development in the whole region (33). The living environment and structure of rural residents are improved, which optimizes the living system of RHSE.

#### 2.3.2 CLC improves the quality of RHSE by promoting livable and moderate living spaces

In response to the issues of inconvenient living space such as scattered layout of rural residential areas, idle homesteads, lack of village construction planning, and shortage of public service facilities, CLC directly affects the living conditions of rural residents by serving their daily, survival, and development needs (11). The efficiency of rural living space allocation is improved, which enhances the living environment in rural areas. This is mainly reflected in enhancing the level of rural residential and service security.

##### 2.3.2.1 CLC improves the quality of RHSE by providing housing security of living space

Construction land consolidation improves the living and social systems of RHSE by providing housing security. Firstly, to increase proportion of public land, by rectifying inefficient or scattered rural homesteads, idle rural construction land, and abandoned industrial and mining construction land, construction land consolidation reorganizes abandoned houses, old facilities, and deteriorating nodes scattered in rural residential areas (31). The reorganization of land optimizes the spatial structure of rural residential areas and meets the housing needs of farmers, and disorderly housing construction in rural areas is rectified (7). At the same time, by promoting the adjustment of land use structure for rural living space construction, the transformation of land use from extensive to intensive has been achieved. The marginalization of rural homesteads, characterized by the weakening of residential security functions and the manifestation of operational functions, has been addressed to a certain extent (4), which improves the residential system of RHSE. Secondly, in conjunction with the policy of withdrawing villages and merging them, construction land consolidation is centralized and unified the living areas of farmers through the consolidation of homestead land through measures such as crisis prevention and demolition, beautification of houses, and greening of courtyards. The interaction between farmers and surrounding neighbors is strengthened, which builds harmonious and friendly neighborhood relationships, and improves the social system of RHSE.

Ecological restoration improves the natural and residential systems of RHSE by providing housing security. Firstly, to increase effective irrigation rate, ecological restoration utilizes projects such as abandoned mine management, green mine construction, soil pollution remediation, and water environment management (33). Ecological restoration promotes comprehensive management of soil erosion and carry out restoration of wildlife habitats (8). The layout of rural ecological land is optimized and adjusted, and the functions of damaged rural ecosystems are protected and restored, which improve the quality of RHSE and enhance the natural system of RHSE. Secondly, to increase proportion of public land, by promoting rural toilet renovation, garbage classification, and sewage treatment, ecological restoration improves the quality of RHSE and increase complete environmental facilities (6). The livability of rural living environment is constantly improving, and the residential system of RHSE is being enhanced.

##### 2.3.2.2 CLC improves the quality of RHSE through improving service security of living space

Construction land consolidation provides service security to improve the supportive system, natural system, and social system of RHSE. Firstly, to increase proportion of public land, construction land consolidation transforms abandoned industrial and mining areas into construction land that serves industrial development, public services, and infrastructure construction (1). This improves the supporting infrastructure for public services and provides various guarantee systems to support human activities. Secondly, to increase road convenience, by constructing and maintaining rural living infrastructure such as sewage, garbage, toilets, roads, and streetlights, construction land consolidation improves the public sanitation facilities in villages and enhances their transportation convenience. The stereotype of dirty, messy, and poor rural areas is changed (7), and the natural system of rural areas is optimized. Thirdly, construction land consolidation provides a vast open space for people to interact with each other in rural areas through the improvement of rural basic public service facilities and leisure and entertainment facilities. The increase in interpersonal communication and social bonds among rural residents is optimized the social system of RHSE (1). Construction land consolidation provides strong support for the improvement of rural living conditions and the efficient implementation of comprehensive rural governance, which promotes the construction of RHSE and improve the quality of life of villagers.

Ecological restoration improves the human system and supportive system of RHSE by providing service security. To increase effective irrigation rate, ecological restoration involves setting up centralized disposal points for garbage and sewage, constructing irrigation reservoirs, dams, drainage channels and other irrigation facilities, as well as production access roads (15), to develop an environmentally friendly and efficient drainage network (33). At the same time, to increase proportion of public land and land use elasticity, by restoring and improving the pattern and connectivity of rural ecological spaces, CLC enhances the convenience of rural residents’ lives and improves corresponding public service facilities. The ecosystem service function is enhanced, optimizing the human system and supportive system of RHSE.

#### 2.3.3 CLC improves the quality of RHSE by ensuring green and sustainable ecological spaces

In response to the deterioration of ecological spaces such as shrinking green spaces, environmental pollution, and fragmented ecological landscapes in rural areas, CLC focuses on the construction of green infrastructure to enhance the ecological viewing and maintenance functions of rural ecological spaces (8). CLC shifts from single factor remediation to multi factor remediation, from land consolidation to regional remediation, and emphasizes that ecological environment is the fundamental condition for rural development.

##### 2.3.3.1 CLC improves RHSE by enhancing the ecological viewing function of ecological space

Agricultural land consolidation improves the natural and human systems of RHSE by enhancing the ecological viewing function of ecological space. Firstly, to increase concentration and connectivity of cultivated land and land reclamation rate, the consolidation of agricultural land improves the natural system of RHSE. On the one hand, based on large-scale agriculture, agricultural land consolidation develops rural landscape resources by utilizing crop resources, agricultural land, and crop production methods (15). The diversity and uniqueness of rural landscapes have increased and the value of rural natural resources has become apparent (4), which improving the natural system of RHSE. On the other hand, through methods such as cultivating paddy fields, demolishing old land for reclamation, and constructing high standard farmland (14), agricultural land consolidation forms a large-scale and interconnected farmland infrastructure network (7), and changes the topography of rural farmland. At the same time, a unique terraced landscape is formed in rural areas through the transformation of slopes into terraces. The ecosystem diversity, stability, and sustainability of rural ecosystems are enhanced (33), and the natural system of RHSE is improved. Secondly, to increase green vegetation coverage rate, through the cultivation of fruit trees, vegetables, flowers, field crops, and medicinal herbs through agricultural land consolidation, species richness and biodiversity in rural areas are increased. This improves the ecological viewing function of rural ecological space, optimizes the rural ecological landscape pattern, and enhances the human system of rural living environment.

Construction land consolidation improves the ecological viewing function of ecological space, enhancing the social system, residential system, and supportive system of RHSE. To increase the green vegetation coverage rate in rural areas, the CLC enhances the coordination between new rural buildings and the overall rural landscape through the consolidation of rural residential areas. It preserves rural cultural characteristics, retains the unique cultural heritage and cultural traits of rural areas, and improves the social system and residential system of RHSE (4, 8, 11).

Ecological restoration improves the natural, social, and human systems of RHSEs by enhancing the ecological viewing function of ecological spaces. Firstly, to increase green vegetation coverage rate, concentration and connectivity of cultivated land and land reclamation rate, by cultivating inefficient abandoned farmland into forests and grasslands, and reasonably greening abandoned industrial and mining land, the ecological viewing function of the ecological space is improved (4), and the natural system of rural living environment is enhanced. Secondly, to increase the diversity of rural landscapes, ecological restoration focuses on the protection and restoration of rural style and landscape, while ensuring the historical and cultural characteristics of rural areas (32). By coordinating cultural and natural landscapes, the unique natural features such as rural landscapes are protected. The cultural context and local memories of rural areas are inherited. By retaining nostalgia, the cultural value and humanistic sentiment of rural areas are enhanced (33). The ecological viewing function of ecological space is improved. On the one hand, the unique cultural genes of rural areas strengthen emotional connections between people. On the other hand, it attracts tourists to visit and strengthens the collective economy. The social and human systems of RHSE are improved (2).

##### 2.3.3.2 CLC improves the quality of RHSE by enhancing ecological maintenance function of ecological space

Agricultural land consolidation improves the natural system of RHSE by enhancing ecological maintenance functions. Firstly, to increase green vegetation coverage rate and concentration and connectivity of cultivated land, by utilizing measures such as planting ecological protective forest nets in farmland, constructing slope protection embankments, and dredging rivers, agricultural land consolidation helps prevent wind and sand erosion, and reduces the risk of soil erosion. The flood and drought resistance of farmland has been improved, and the harm of droughts and floods to rural areas has been reduced (4). The ecological maintenance function of rural areas is improved. The natural system of RHSE is perfected. Secondly, to increase land reclamation rate, by implementing land leveling projects and field road construction, the consumption of fertilizers and pesticides during use is reduced to a certain extent (33). The extensive land use in rural areas is improved. The soil organic matter content is increased. The vegetation survival rate and landscape diversity are increased, and the natural system of RHSE is optimized.

The consolidation of construction land enhances the ecological maintenance function and improves the natural system of RHSE. By improving the ecological maintenance function, the natural system of RHSE is perfected. Firstly, to increase green vegetation coverage rate and the diversity of rural landscapes, by rectifying idle and underutilized construction land, the “hollow villages” are demolished and reclaimed, resulting in forests and grasslands. The increase in forest and grassland areas (8). The improvement of rural vegetation coverage highlights the value of windbreak, sand fixation, and water conservation in rural ecological service systems (30). Secondly, abandoned or underutilized ponds in rural areas are converted into ecological green spaces. The spatial layout of rural areas is reorganized. Meanwhile, the diversity of rural landscapes is increased through the cultivation of ornamental flowers, waterfront landscapes, and the construction of green belts. The ecological maintenance function of rural areas is enhanced, and the natural system of RHSE is improved.

Ecological restoration improves the natural system of RHSE by enhancing ecological maintenance functions. Ecological restoration focuses on enhancing the ecological maintenance function of natural environments such as forests, grasslands, wetlands, and water bodies. Firstly, to increase green vegetation coverage rate, ecological restoration involves the restoration and management of water bodies such as rural rivers, reservoirs, and groundwater, as well as the delineation of protection red lines for rural forests (32). The pattern of ecological spaces such as rural water systems and forest networks is optimized (33). At the same time, rural roads are comprehensively greened and developed into forests or belts according to local conditions (4), increasing the coverage of rural vegetation. The natural foundation of rural areas is restored, improving the natural system of RHSE. Secondly, to increase the diversity of rural landscapes, ecological restoration relies on measures such as increasing the number of trees on slopes and planting protective forests to prevent soil erosion and protect rural habitats. The vitality of rural flora and fauna is enhanced and biodiversity is improved, which is conducive to building a healthy and orderly biological network and ensuring its sustainable operation (32, 33). At the same time, ecological corridors and biodiversity conservation networks are constructed through highland reinforcement and slope improvement. The rural ecological security barrier system has been optimized to build a stable and sustainable ecological security pattern. The benign collaboration and circulation among ecological elements are promoted, strengthening rural ecological resilience and optimizing the natural system of RHSE.

## 3 Research methods

Firstly, this article constructed a CLC level and RHSE quality evaluation system, taking Zhejiang Province as an example, to explore the mechanisms of CLC on RHSE. The entropy method was used to measure the weight of indicators. CLC level and RHSE quality of Zhejiang Province and various prefecture level cities were evaluated in the year of 2000, 2005, 2010, 2015, and 2020. Secondly, a geographic detector model was constructed for factor detection and factor interaction detection to explore the explanatory power and interactive effects of various CLC factors on the quality of RHSE.

### 3.1 Evaluation index system for RHSE quality

According to Wu’s classification, the living environment is divided into five aspects: natural, human, residential, social, and supportive. This study selects indicators including plastic film coverage area, agricultural fertilizer application intensity, biological richness index, rural labor level, and gender balance rate (3, 15, 20, 21, 36), constructing an evaluation index system for RHSE quality (Table 2).

**Table 2:** Evaluation index system and data sources for RHSE quality.

First level indicators	Second level indicators	Third level indicators	Direction	Explain	Data source	Indicators weight
Rural human settlement environment (RHSE)	Natural	Plastic film coverage area	Negative	/	Zhejiang Statistical Yearbook, Zhejiang Provincial Statistical Yearbook of Natural Resources and Environment, and statistical yearbooks of various districts (counties, cities)	0.059
		Agricultural fertilizer application rate	Negative	Agricultural fertilizer application rate/Cultivated area	Zhejiang Statistical Yearbook and statistical yearbooks of various districts (counties, cities)	0.051
		Biological richness index	Positive	$\begin{array}{l} D=0.35\times S_{1}+0.21\times S_{2}+0.28\times S_{3}+\\ 0.11\times S_{4}+0.04\times S_{5}+0.01\times S_{6} \end{array}$	Zhejiang Statistical Yearbook, Zhejiang Provincial Statistical Yearbook of Natural Resources and Environment, and statistical yearbooks of various districts (counties, cities)	0.061
	Human	Rural labor force level	Positive	Rural labor force quantity/Total rural population	Zhejiang Statistical Yearbook and statistical yearbooks of various districts (counties, cities)	0.059
		Gender balance rate	Negative	(Male population - Female population)/Total rural population	Zhejiang Provincial Population Census Data and population census data of various districts (counties, cities)	0.053
		Per capita education, culture and entertainment expenditure of rural residents	Positive	/	Zhejiang Statistical Yearbook and statistical yearbooks of various districts (counties, cities)	0.059
		Family size	Positive	Total rural population/ Total number of rural households	Zhejiang Provincial Population Census Data and population census data of various districts (counties, cities)	0.057
	Residential	Housing area	Positive	/	Zhejiang Statistical Yearbook and statistical yearbooks of various districts (counties, cities)	0.060
		Proportion of rural residents receiving education	Positive	Number of educated villagers in rural areas/Total rural population	Zhejiang Statistical Yearbook and statistical yearbooks of various districts (counties, cities)	0.058
		Average color TV ownership per 100 households in rural areas	Positive	/	Zhejiang Statistical Yearbook and statistical yearbooks of various districts (counties, cities)	0.057
		Number of hospital and health center beds	Positive	/	China Social Statistical Yearbook, Zhejiang Statistical Yearbook, and statistical yearbooks of various districts (counties, cities)	0.063
	Supportive	Average mobile phone ownership per 100 households in rural areas	Positive	/	Zhejiang Statistical Yearbook and statistical yearbooks of various districts (counties, cities)	0.058
		Per capita water resources	Positive	Total water resources/Total rural population	Zhejiang Statistical Yearbook and statistical yearbooks of various districts (counties, cities)	0.062
		Rural power facility level	Positive	Rural electricity consumption/Total rural population	Zhejiang Statistical Yearbook and statistical yearbooks of various districts (counties, cities)	0.063
	Social	Agricultural economic development rate	Positive	Total output value of agriculture, forestry, animal husbandry and fishery /Total rural population	Zhejiang Statistical Yearbook and statistical yearbooks of various districts (counties, cities)	0.064
		Urban–rural per capita income ratio	Negative	Disposable income of urban residents/Disposable income of rural residents	Zhejiang Statistical Yearbook and statistical yearbooks of various districts (counties, cities)	0.053
		Ratio of social security expenditure to GDP	Positive	Social security expenditure/ Regional GDP	Zhejiang Statistical Yearbook and statistical yearbooks of various districts (counties, cities)	0.063

The determination of the weight of factors affecting the biological richness index is based on the “Technical Specifications for Ecological Environment Status Assessment” (HJ/T192-2015). Among them, $S_{1}$ represents the proportion of forest land area; $S_{2}\;$represents the proportion of grassland area; $S_{3}$ represents the proportion of water and wetland area; $S_{4}$ represents the proportion of cultivated land area; $S_{5}$ represents the proportion of construction land area; $S_{6}$ represents the proportion of unused land area; GDP represents the gross domestic product.

### 3.2 Evaluation index system for CLC

According to the Production-Living-Ecological Space theory, selected indicators including high standard farmland area, average grain yield per mu, proportion of public land, and road convenience from three aspects: production, living, and ecology (8, 14, 15, 26, 30, 35, 37, 38). The evaluation index system for CLC is constructed (Table 3).

**Table 3:** Evaluation index system and data sources for CLC.

First level indicators	Second level indicators	Third level indicators	Direction	Explain	Data source	Indicators weight
Comprehensive land consolidation (CLC)	Production	High standard farmland area (X_1_)	Positive	Basic farmland area	China Natural Resources Statistical Yearbook, village plans of various districts (counties, cities) in Zhejiang Province, and basic farmland area data in government work reports of various districts (counties, cities)	0.076
		Average grain yield per mu (X_2_)	Positive	Total grain production/Total cultivated land area for planting grain	Data on total grain production was from China Rural Statistical Yearbook and grain production data in statistical yearbooks of various districts (counties, cities). Data on total cultivated land area for planting grain was from Zhejiang Statistical Yearbook and data on grain sown area in statistical yearbooks of various districts (counties, cities).	0.082
		Per capita net income of villagers (X_3_)	Positive	/	Statistical Bulletin of National Economic and Social Development of Zhejiang Province and statistical bulletins of national economic and social development of various cities	0.084
		Net output value of grain per mu (X_4_)	Positive	output value of grain crops -Personal input of farmers	China Agricultural Machinery Industry Yearbook	0.079
	Living	Proportion of public land (X_5_)	Positive	Total area of public land/Total area of land in the project area	China Urban and Rural Construction Statistical Yearbook and survey data	0.089
		Road convenience (X_6_)	Positive	Road area/Total land area of the project area	China Urban and Rural Construction Statistical Yearbook and survey data	0.088
		Land use elasticity(X_7_)	Positive	Blank land/Total area of land in the project area	Main data bulletins of the national land survey and survey data of Zhejiang Province and various districts (counties, cities)	0.081
		Effective irrigation rate(X_8_)	Positive	Total area of paddy fields and irrigated land/Total area of cultivated land	Village plans of various districts (counties, cities) in Zhejiang Province, government work reports of various districts (counties, cities), public data from Zhejiang Provincial Department of Natural Resources	0.089
	Ecology	Green vegetation coverage rate(X_9_)	Positive	Sum of green plant area/Total land area of the project area	China Urban and Rural Construction Statistical Yearbook and survey data	0.083
		Concentration and connectivity of cultivated land(X_10_)	Positive	Total area of cultivated land/Total number of cultivated land blocks	Village plans of various districts (counties, cities) in Zhejiang Province, government work reports of various districts (counties, cities), public data from Zhejiang Provincial Department of Natural Resources	0.084
		Land reclamation rate(X_11_)	Positive	Total cultivated land area/Total land area of the project area	Village plans of various districts (counties, cities) in Zhejiang Province, government work reports of various districts (counties, cities), public data from Zhejiang Provincial Department of Natural Resources	0.088
		Shannon’s Diversity Index (SHDI) (X_12_)	Positive	$\text{SHDI}=-\sum_{i=1}^{m}(p_{i}\ln p_{i})$ M is the land use type, $p_{i}$ represents the proportion of land use type i in the study area.	Institute of Geographic Sciences and Natural Resources Research, Chinese Academy of Sciences (spatial resolution: 30 m). https://www.resdc.cn (accessed on 18 June 2023)	0.077

### 3.3 Entropy method

To eliminate the impact of different dimensions among indicators on the evaluation results, all indicators were standardized. Subsequently, entropy method was used to determine the indicator weights (39).

### 3.4 Geographic detector

The geographic detector was employed to examine the influence mechanism of various factors on RHSE. The geographic detector model is immune to multicollinearity, avoiding endogeneity issues where independent and dependent variables are mutually causal, and exploring the spatial differentiation characteristics of each input factor from a heterogeneity perspective (Equation 1) (40).


(1) $$q_{j}=1-\frac{\sum_{h=1}^{L}N_{\mathrm{jh}}\sigma_{\mathrm{jh}}^{2}}{N_{j}\sigma_{j}^{2}}$$

In the above equation, h = 1, L is the partition (classification) number of the influencing factor j; $N_{\mathrm{jh}}\;\text{and}\;N_{j}$ represents the interval (class) h of the influencing factor j and the number of units in the entire region; $\sigma_{j}^{2}$ and $\sigma_{\mathrm{jh}}^{2}$ represents the sum of the total variance of the entire region and the variance within the interval (class). $q_{j}$ is the strength of the impact of factor j on the spatial heterogeneity of RHSE; $q_{j}\in [0,1]$, the larger the $q_{j}$, the higher the degree of impact. The interaction detection of geographic detectors can be achieved by examining whether the strength of the effects of multiple independent variables on the dependent variable is enhanced relative to the effect of a single independent variable on the dependent variable. *P* Value test whether the influencing factor j has an impact on the RHSE. There are five types of effects of two dependent variables:

(1) $q(x_{1}\cap x_{2})<\min(q(x_{1}),q(x_{2}))$ indicates that the interaction between $x_{1}$ and $x_{2}$ shows a nonlinear weakening;
(2) $q(x_{1}\cap x_{2})>\max(q(x_{1}),q(x_{2}))$ indicates that the interaction between $x_{1}$ and $x_{2}$ shows a dual factor enhancement;
(3) $\min(q(x_{1}),q(x_{2}))<q(x_{1}\cap x_{2})<\max(q(x_{1}),q(x_{2}))$ indicates that the interaction between $x_{1}$ and $x_{2}$ shows a single factor nonlinear weakening;
(4) $q(x_{1}\cap x_{2})>q(x_{1})+q(x_{2})$ indicates that the interaction between $x_{1}$ and $x_{2}$ exhibits nonlinear enhancement;
(5) $q(x_{1}\cap x_{2})=q(x_{1})+q(x_{2})$ indicates that $x_{1}$ and $x_{2}$ are independent of each other.

## 4 Results

### 4.1 Development trend of RHSE and CLC

**Figure 4:**
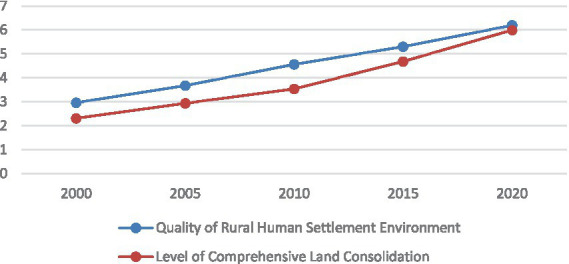
The level of CLC and the evolution of RHSE quality in Zhejiang Province.

At the provincial level, t the development of RHSE and CLC in Zhejiang Province exhibited an upward trend throughout the entire research period. From 2000 to 2020, the CLC level has improved significantly across the entire region. With the reintegration of the three living spaces, the quality of RHSE has been significantly improved (Figure 4).

**Figure 5:**
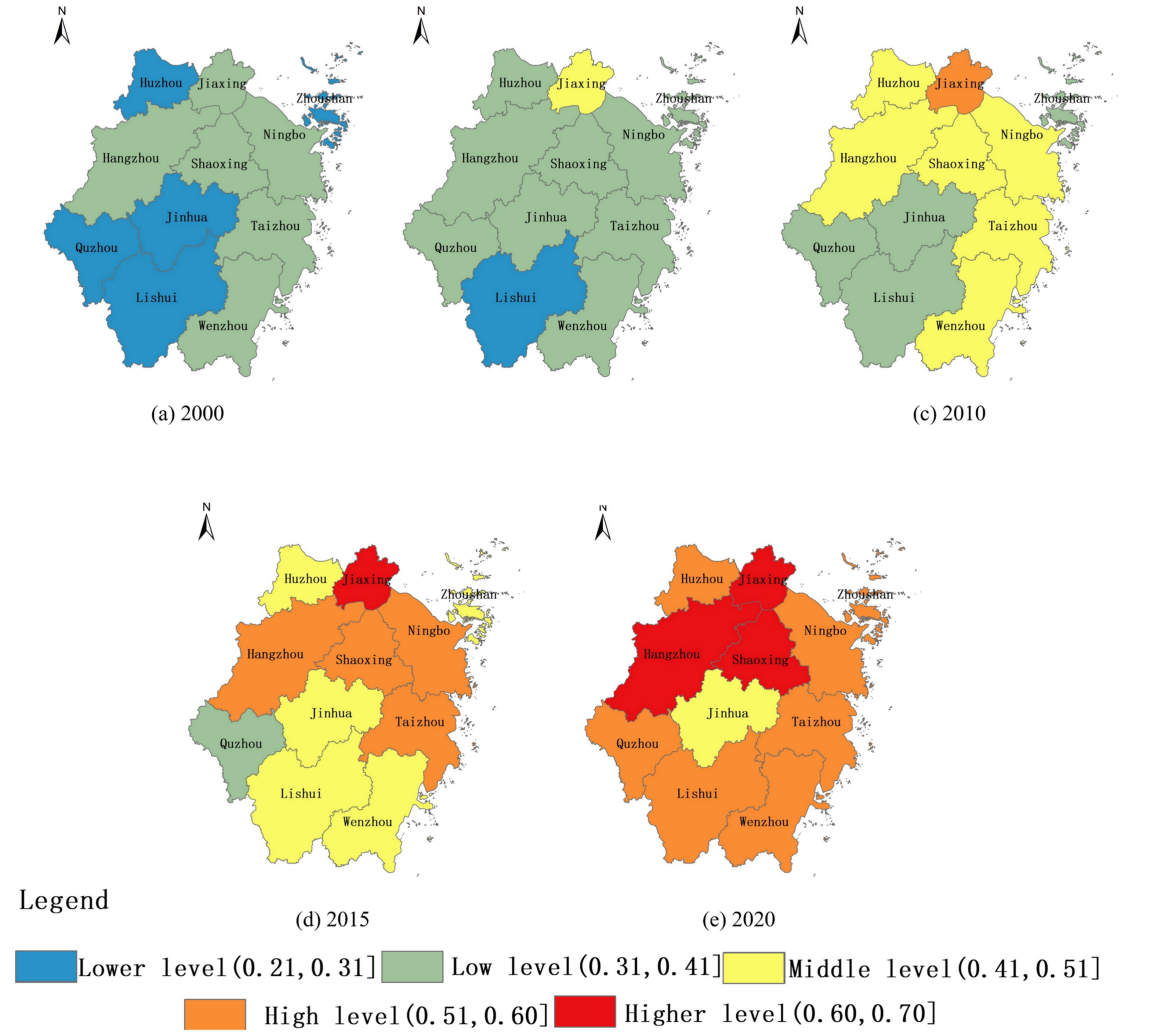
Evolution of RHSE quality in Zhejiang Province. **(a)** 2000 denotes the RHSE quality in the year 2000; **(b)** 2005 denotes the RHSE quality in the year 2005; **(c)** 2010 denotes the RHSE quality in the year 2010; **(d)** 2015 denotes the RHSE quality in the year 2015; **(e)** 2020 denotes the RHSE quality in the year 2020.

Using the entropy method, the human settlements quality of each evaluation unit was calculated based on the evaluation index system of RHSE quality (Table 2). The RHSE quality in 2000, 2005, 2015, and 2020 was classified in accordance with the natural breaks classification standard of 2010. In 2000, the overall quality of RHSE across prefecture level cities in Zhejiang Province was very low. However, in the past 20 years of development, the quality of RHSE had significantly improved (Figures 5A–E). In 2015, there was a high-level integration of RHSE centered on cities such as Jiaxing, Ningbo, Hangzhou, Shaoxing, Taizhou. In 2020, the improvement of RHSE quality in various cities in Zhejiang Province was more significant, with all cities except Jinhua being at a high level.

**Figure 6:**
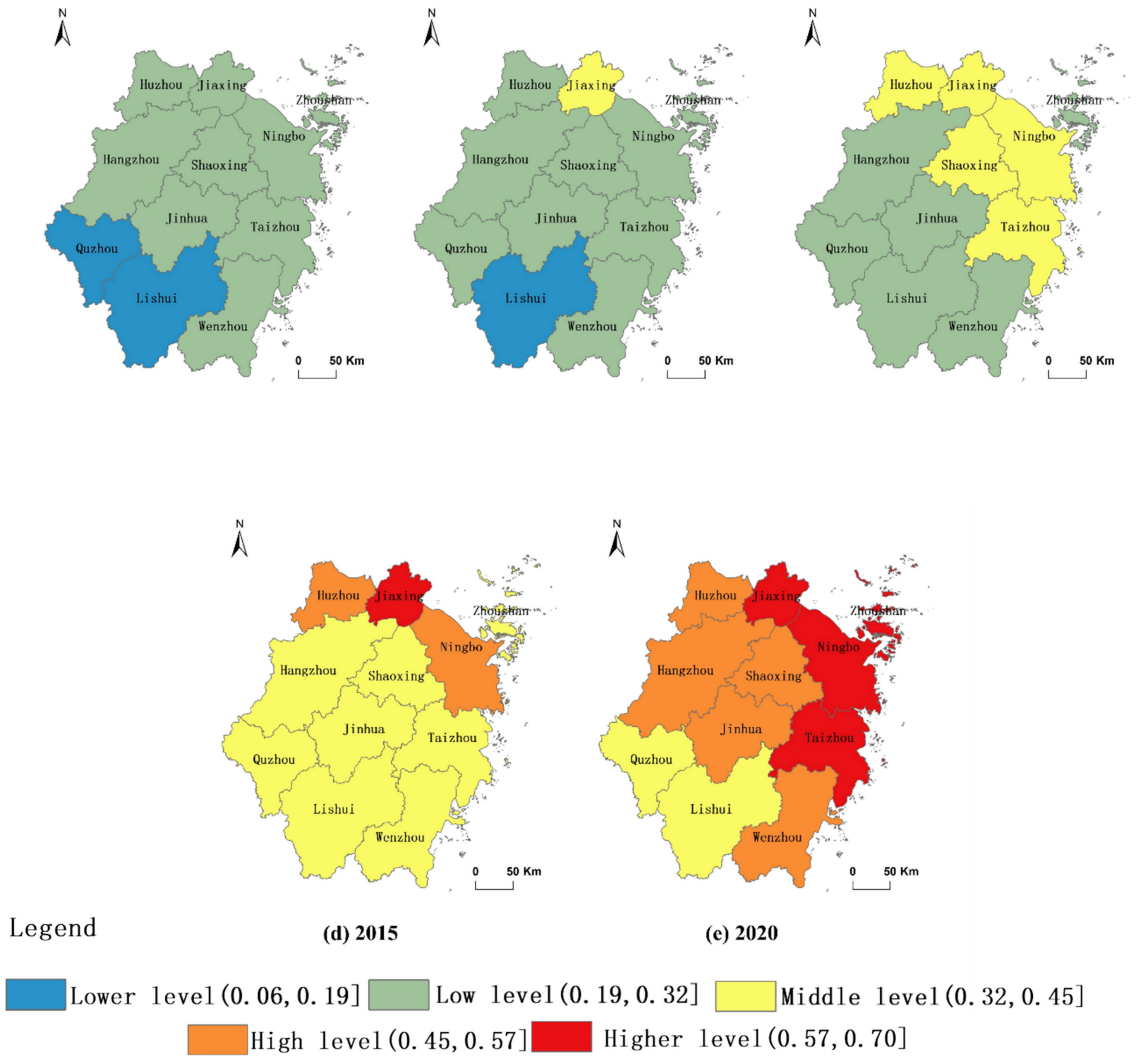
Evolution of CLC level in Zhejiang Province. **(a)** 2000 denotes the CLC level in the year 2000; **(b)** 2005 denotes the CLC level in the year 2005; **(c)** 2010 denotes the CLC level in the year 2010; **(d)** 2015 denotes the CLC level in the year 2015; **(e)** 2020 denotes the CLC level in the year 2020.

Using the entropy method, the CLC level of each evaluation unit was calculated based on the evaluation index system of CLC level (Table 3), and classified using natural breaks. The CLC level of all cities in Zhejiang Province has significantly improved after 2010 (Figure 6). Spatial consistency was observed between the evolutionary processes of RHSE and CLC levels. The evaluation results indicate that CLC plays a key role in improving the RHSE by driving rural spatial reconstruction and optimizing the functions of the three living spaces.

### 4.2 Assessment of the impact of CLC on RHSE

RHSE was the dependent variable in this study. The independent variables were high standard farmland area, average grain yield per mu, per capita net income of villagers, average net grain output per mu, proportion of public land, road convenience, land use elasticity, effective irrigation rate, green vegetation coverage, concentration and contiguous farmland, land reclamation rate, and landscape diversity. Geographic detectors were employed to measure the impact of different indicators on RHSE. The research results showed that different dimensions and indicators had varying degrees of impact on RHSE in Zhejiang Province.

#### 4.2.1 Single factor detection

Using the single factor detection model of the geographical detector, the influence magnitude of each indicator was calculated. The single factor detection results indicated differences in the single-factor impact of comprehensive land consolidation across different years (Table 4). However, the highest impact factor was highly consistent. In 2000, the top six highest single factor rankings from large to small were X_2_, X_10_, X_7_, X_8_, X_9_, and X_11_. In 2005, the top six highest single factor rankings from large to small were X_11_, X_2_, X_10_, X_7_, X_12_, and X_4_. In 2010, the top six highest single factor rankings from large to small were X_5_, X_11_, X_10_, X_8_, X_2_, and X_7_. In 2015, the top six highest single factor rankings from large to small were X_11_, X_8_, X_10_, X_5_, X_4_, and X_3_. In 2020, the top six highest single factor rankings from large to small were X_11_, X_8_, X_10_, X_12_, X_5_, X_3_. Overall, the single factor detection results indicated that X_2_, X_5_, X_7_, X_8_, X_10_, and X_11_ were the most important CLC factors that had an impact on RHSE. It can be seen that CLC has a high explanatory power for improving the quality of RHSE, by promoting the increases in average grain yield per mu, public land area, land use elasticity, effective irrigation rate, concentrated and contiguous farmland, land reclamation rate, etc.

**Table 4:** Single factor detection results (*q*-value).

Variables year	X_1_	X_2_	X_3_	X_4_	X_5_	X_6_	X_7_	X_8_	X_9_	X_10_	X_11_	X_12_
2000	0.259***	0.751***	0.219***	0.176***	0.202***	0.174***	0.473***	0.420***	0.280***	0.483***	0.261***	0.231***
2005	0.254***	0.521***	0.207***	0.355***	0.145***	0.296***	0.382***	0.196***	0.202***	0.403***	0.619***	0.375***
2010	0.066***	0.391***	0.019	0.236***	0.608***	0.025***	0.368***	0.443***	0.124***	0.469***	0.469***	0.139***
2015	0.112***	0.078***	0.385***	0.389***	0.395***	0.043***	0.057***	0.613***	0.110***	0.445***	0.626***	0.105***
2020	0.308***	0.110***	0.421***	0.314***	0.510***	0.412***	0.379***	0.738***	0.218***	0.614***	0.769***	0.543***

(a) *, **, *** represent the significance levels of each factor detection result at 0.1, 0.05, and 0.01, respectively. (b) X_1_ indicates the high standard farmland area; X_2_ indicates the average grain yield per mu; X_3_ indicates the per capita net income of villagers; X_4_ indicates the net output value of grain per mu; X_5_ indicates the proportion of public land; X_6_ indicates the road convenience; X_7_ indicates the land use elasticity; X_8_ indicates the effective irrigation rate; X_9_ indicates the green vegetation coverage rate; X_10_ indicates the concentration and connectivity of cultivated land; X_11_ indicates the land reclamation rate; X_12_ indicates the Shannon’s diversity index (SHDI).

#### 4.2.2 Factor interaction detection

**Figure 7:**
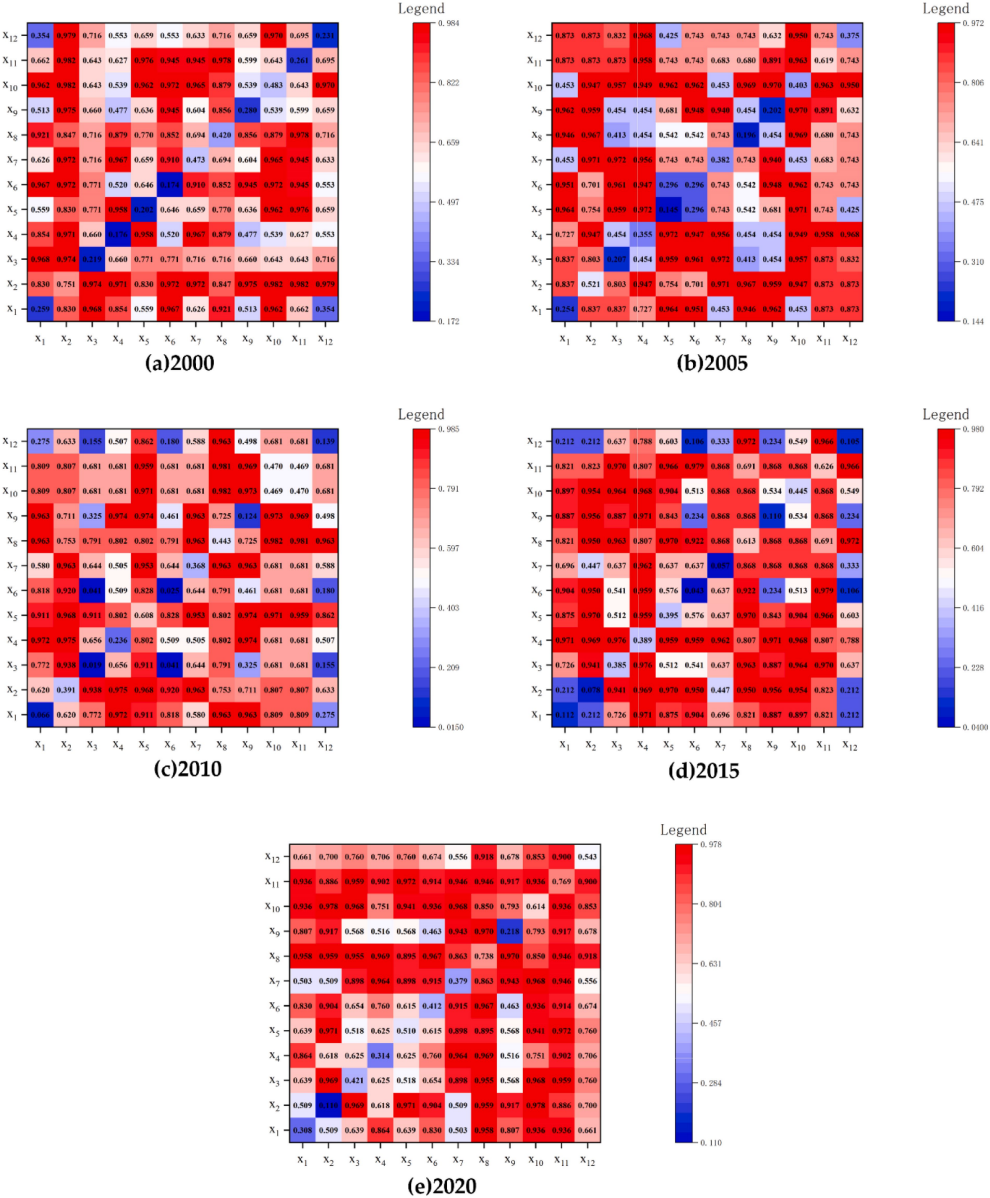
Interaction of CLC factors(q). Notes: X_1_ indicates the high standard farmland area; X_2_ indicates the average grain yield per mu; X_3_ indicates the per capita net income of villagers; X_4_ indicates the net output value of grain per mu; X_5_ indicates the proportion of public land; X_6_ indicates the road convenience, X_7_ indicates the land use elasticity; X_8_ indicates the effective irrigation rate; X_9_ indicates the green vegetation coverage rate; X_10_ indicates the concentration and connectivity of cultivated land; X_11_ indicates the land reclamation rate; X_12_ indicates the Shannon’s diversity index (SHDI). **(a)** 2000 denotes the interaction of CLC factors(q) in the year 2000; **(b)** 2005 denotes the interaction of CLC factors(q) in the year 2005; **(c)** 2010 denotes the interaction of CLC factors(q) in the year 2010; **(d)** 2015 denotes the interaction of CLC factors(q) in the year 2015; **(e)** 2020 denotes the interaction of CLC factors(q) in the year 2020.

Using the factor interaction detection model of the geographical detector, the magnitude of influence of the interactions between indicators on rural human settlements was calculated. The analysis of the interactive effects of CLC is helpful for further understanding its driving mechanism on RHSE. Based on detecting 12 single factors, this article explored the interaction between CLC factors. The detection results indicated that the interaction q-values of different CLC factors are greater than those of individual factors, exhibiting dual factor enhancement or nonlinear enhancement effect. The results indicated that the impact of all CLC factors on RHSE was not independent. In other words, the interaction between factors was higher than the impact of a single factor on RHSE. The interaction between individual factors can better explain the regional differences in RHSE. But there were differences in the impact of the interaction of different influencing factors on RHSE (Figure 7).

In 2000, X_1_, X_2_, X_3_, X_5_, X_6_, X_7_, X_8_, X_10_, and X_11_ exhibited strong interactions with other factors, and the average q-value of factor interaction detection was greater than 7, with an explanatory power of exceeding 70%. The strongest interactions were between X_2_ and other factors, with an average q-value greater than 8 for factor interaction detection and an explanatory power of over 80%.

In 2005, except for X_5_ and X_7_, the average q-value of factor interaction detection with other factors was greater than 0.7, with an explanatory power of over 70%. The stronger interactions were between X_2_, X_10_, X_11_ and other factors, with an average q-value greater than 8 for factor interaction detection and an explanatory power of over 80%.

In 2010, the interaction between X_1_, X_2_, X_5_, X_7_, X_8_, X_9_, X_10_, X_11_ and other factors was strong. The average q-value of factor interaction detection was greater than 7, with an explanatory power of over 70%. The higher factors were the interaction between X_5_ and X_8_ with other factors, with an average q-value greater than 8 for factor interaction detection and an explanatory power of over 80%.

In 2015, the interaction between X_2_, X_3_, X_4_, X_5_, X_8_, X_10_, X_11_ and other factors was strong. The average q-value of factor interaction detection was greater than 7, with an explanatory power of over 70%. The stronger interactions were between X_4_, X_8_, X_11_ and other factors, with an average q-value greater than 8 for factor interaction detection and an explanatory power of over 80%.

In 2020, except for X_9_, the average q-value of factor interaction detection between all factors and other factors was greater than 7, with an explanatory power of over 70%. The higher factors were the interaction between X_8_, X_10_, X_11_ and other factors, with an average q-value greater than 8 for factor interaction detection and an explanatory power of over 80%.

Overall, the q values of interactions between X2, X10, X11, and other factors were relatively high throughout the study period. The explanatory power of interactions between X8, X11, and other factors on RHSE quality increased significantly. The results revealed the important role of CLC in improving the average grain yield per mu, effective irrigation rate, concentrated contiguous farmland, and land reclamation rate in promoting the development of RHSE.

#### 4.2.3 Causal analysis

The single factor detection results indicated that the average grain yield per mu(x_2_), the proportion of public land(x_5_), the land use elasticity(x_7_), the effective irrigation rate(x_8_), the concentration and connectivity of cultivated land(x_10_), and the land reclamation rate(x_11_) were the most important CLC factors that affected the RHSE. The interaction between the average grain yield per mu(x_2_), effective irrigation rate(x_8_), concentration and connectivity of cultivated land(x_10_), and land reclamation rate(x_11_) and other factors were strong relatively, indicating that these indicators had a strong effect on improving RHSE when combined with other factors. Therefore, CLC had a significant impact on RHSE by increasing the average grain yield per mu, the proportion of public land, land use elasticity, effective irrigation rate, and land reclamation rate. The main reasons are as follows.

Firstly, by constructing agricultural water conservancy projects and field road projects, and equipping them with corresponding irrigation and drainage facilities, the effective irrigation rate and land use elasticity were improved, resulting in the improvement of the natural and supporting systems of RHSE. Secondly, arable land was fundamental to the livelihood of rural residents. It was the basic source of livelihood for rural residents. Agricultural land consolidation involved the implementation of land leveling projects that merge small plots into large plots, as well as corresponding measures to adjust land ownership, resulting in the contiguous development and redistribution of fragmented and dispersed farmland. The average grain yield per mu, land reclamation rate, and concentrated contiguous farmland per mu were increased, improving the human and supporting systems of RHSE. Finally, the effective promotion of large-scale, mechanized agricultural development by improving modern facilities during the process of agricultural land consolidation. The efficiency of grain production was improved. The land use patterns was optimized. The agricultural production and food security functions of rural production space were strengthened, improving the human system of RHSE.

Secondly, CLC improved RHSE quality through construction land consolidation in Zhejiang Province. Construction land consolidation involved the consolidation of idle and inefficient construction land, the relocation of scattered farmhouses scattered in farmland, and the reclamation of abandoned homesteads. On the one hand, it promoted the concentration of cultivated land and increases the average grain yield per mu, which improved the human system of RHSE. On the other hand, the surplus construction land quotas were used for public infrastructure and housing construction, increasing the proportion of public land. The increase of land used for education, healthcare, and other purposes improved the social and residential systems of RHSEs.

Thirdly, CLC improved the quality of RHSE through ecological restoration in Zhejiang Province. Firstly, the farmland ecosystem protection project was implemented to carry out ecological restoration. On the one hand, the soil fertility of cultivated land was improved, while the average grain yield per mu and land use elasticity were increased, which improved the natural and supporting systems of RHSE. On the other hand, ecological restoration was combined with rural ecological tourism to maintain biodiversity and construct ecological landscape patterns. Farmers’ incomes increased and the proportion of public land rose. RHSE supportive system, social system, and residential system were improved.

## 5 Discussion and policy recommendations

The results of this study indicate that the RHSE has improved year by year, which is consistent with the findings of Cheng et al., Wu et al., and Lin and Hou (1, 21, 41). Cheng et al. argued that the quality of China’s rural environment shows an annual upward trend: from eliminating squalor and disorder to pursuing rural aesthetics, significant achievements have been made in improving China’s rural human settlement environment. Wu et al. maintained that the human settlement environment index has increased significantly across all 30 provinces in China. Lin et al. suggested that the improvement of the RHSE in Zhejiang Province has promoted the sustainable development of rural areas.

Meanwhile, the study results demonstrate that CLC has enhanced the RHSE, which aligns with the research findings of Huang et al. (42)and Zhou et al. (5). Huang et al. proposed that the consolidation of rural residential areas is essentially a process of optimizing agricultural production, livelihoods, and space. Therefore, by adjusting the current spatial distribution and structure of these residential areas, rural living conditions—including those related to the economy and ecology—can be greatly improved. Zhou et al. noted that in recent years, with the growing attention paid to environmental issues in China, land consolidation not only needs to meet the requirements of increasing the quantity and improving the quality of cultivated land but has also been endowed with new functions such as improving the human settlement environment, supporting poverty alleviation, and revitalizing rural areas.

Going beyond existing studies, this research analyzes the evolution of both CLC and the RHSE, while quantitatively examining the impact of various factors of CLC on the RHSE. This enables the accurate identification of the impact of each CLC factor on the RHSE. Furthermore, land consolidation at the current stage tends to be implemented in a goal-oriented manner. As two key components of rural revitalization initiated by the Chinese government, verifying the policy effects of CLC and promoting the continuous optimization of the RHSE are feasible.

### 5.1 Development trend of RHSE and CLC level

#### 5.1.1 Provincial level

CLC level in Zhejiang Province had significantly improved from 2000 to 2020. Under the reorganization of production-living-ecological space, the quality of RHSE in Zhejiang Province had significantly improved. This was attributed to policy promotion in Zhejiang Province. In 1999, land consolidation entered a stage of comprehensive promotion and implementation in Zhejiang Province. The central content of the work was rural land consolidation, mainly including farmland consolidation and land consolidation for rural residential construction. In 2001, exploration of pilot work on linking urban–rural construction land increases and decreases, with rural construction land reclamation as the main content, began in Zhejiang Province. This marked the beginning of comprehensive rural land consolidation in Zhejiang. In June 2003, the Zhejiang Provincial Party Committee launched the Green Rural Revival Program in Zhejiang Province. Zhejiang selected approximately 10,000 administrative villages from 40,000 villages in the province for comprehensive renovation. Approximately 1,000 central villages were developed into comprehensive well-off demonstration villages in Zhejiang. In 2017, he concept of “national comprehensive land consolidation” was officially proposed for the first time in Zhejiang Province. In 2018, in order to form a new pattern of rural land use with contiguous farmland and clustered villages, a regional CLC workstation was established in Zhejiang based on the experience of the Green Rural Revival Program. In September 2018, the “Green Rural Revival Program” was awarded the highest environmental honor by the United Nations Environment Programme-Champions of the Earth Award.

#### 5.1.2 Municipal level

From 2000 to 2020, the CLC levels were relatively high in Hangzhou, Jiaxing and Ningbo. Meanwhile, these cities were also located in areas with a high level of RHSE integration. The results indicated that the CLC in these areas had a significant promoting effect on the RHSE.

CLC was highly valued in Hangzhou, one of the earliest cities in Zhejiang Province to carry out this work. Hangzhou has been implementing large-scale land consolidation since 1999. From 1999 to 2002, land consolidation was in its initial stage in Hangzhou. During this period, the main goal in Hangzhou was to increase arable land area and achieve a balance between arable land occupation and compensation. From 2003 to 2009, it was the initial stage of the “Ten Million Project” in Hangzhou. During this period, land consolidation in Hangzhou was an important part of implementing the “Hundred Village Demonstration and Thousand Village Consolidation” project, and land consolidation was extended from supplementing arable land to renovating villages. From 2010 to 2016, it was the stage of CLC in Hangzhou. During this period, the main policy tool in Hangzhou was to link the increase and decrease of urban and rural construction land. The integration of land development, consolidation, and reclamation has been coordinated, and CLC had been implemented. The diversification of land consolidation goals and implementation models, along with the comprehensiveness of content and benefits, gradually emerged. From 2017 to 2020, Hangzhou was in the stage of CLC. Pilot CLC projects characterized by the comprehensive improvement of “mountains, waters, forests, fields, lakes, roads, and villages” were launched. The transformation of the scope of rectification from scattered land development and consolidation to centralized and contiguous comprehensive rectification has been achieved. Through a series of CLC policies, the RHSE level of Hangzhou has been improved.

In Jiaxing, since the Ministry of Natural Resources supported Zhejiang Province in carrying out comprehensive land consolidation and ecological restoration pilot work, 5 national pilot projects and 85 provincial projects were approved in Jiaxing City. Firstly, funds were concentrates to optimize the overall layout of rural production, living, and ecological spaces. The improvement of RHSE were promoted through comprehensive, multifaceted, and high-efficiency rectification. Secondly, village layout planning was attached great importance in Jiaxing. Through the preparation and adjustment of three rounds of village layout planning, Jiaxing has achieved the agglomeration of rural houses and the optimization of the scale of urban and rural construction land. At the same time, the surveys on the potential of land consolidation was conducted to understand the situation of construction land reclamation, land development, and cultivated land quality in Jiaxing. Thirdly, centralized and contiguous construction of permanent basic farmland within the comprehensive land consolidation project area. The rural reconstruction within project areas was emphasized and strengthened. Efforts were made to address the issue of fragmented arable land and improve its concentration and connectivity. By improving field roads and water conservancy facilities, an ecological irrigation model was formed. The above CLC methods had a positive effect on improving the RHSE in Jiaxing.

In Ningbo, firstly, construction land scale was determined based on overall township land use planning, which was linked to industrial development plans. Based on the development demands of farmers, village level land use plans as the basis for CLC and ecological restoration implementation were formulated. Secondly, CLC was integrated with farmland protection, industrial development, beautiful rural construction, environmental governance, ecological restoration, promoting rural revitalization. The above methods had a positive effect on improving the RHSE.

### 5.2 Policy recommendations

Based on the research findings, the following policies can be implemented to improve the quality of RHSE through CLC.

(1) Integrating high-standard farmland construction and enhancing potential.

Developing high-standard farmland is an essential approach to achieve the goal of building a strong agricultural country. High standard farmland construction is not only the adjustment of farmland structure, but also the driving force for the transformation and upgrading of agricultural production methods and the improvement of ecological environment. Therefore, in the process of agricultural land consolidation, high standard basic farmland construction can be combined. It can not only increase land productivity, but also effectively protect other land ecosystems by reducing blind expansion of planting areas. Firstly, the requirements of basic farmland protection and high standard farmland construction should be aligned with each region, as well as the planning of national land space, modernization of agriculture and rural areas, and water conservancy. The construction layout in various regions is optimized. Secondly, scientifically arrange the timing of high standard farmland construction. The construction in each area should be ensured to be equally effective, thereby improving the transfer rate of arable land in each region. The local crop planting structure is adjusted to ensure increased and stable grain production. The collective economic benefits in rural areas are increased, achieving the goal of increasing farmers’ incomes through industrial development. Finally, local governments should guide farmers to use more scientific methods to improve soil fertility, thereby effectively reducing the use of agricultural production materials such as fertilizers and pesticides that pose certain risks. The government encourages the development of green agriculture in rural areas and promotes the optimization of the social and natural systems of RHSE.

(2) Promoting industrial chain extension and advancing infrastructure development.

Firstly, extending the industrial chain in the process of CLC. Firstly, Through the close integration of agricultural production, processing, and sales via land consolidation, the added value of agricultural products can be significantly increased. Secondly, with the help of construction land consolidation, the government should build agricultural product processing parks and develop the deep processing industry of agricultural. The integration of local agriculture, processing, and service industries should form a complete industrial chain. Thirdly, tourism should be developed in conjunction with the unique ecological landscape of rural areas in the process of ecological restoration, fully leveraging landscape value. The extension of the industrial chain will provide more employment opportunities for farmers and increases their income. The human and supportive systems of RHSE will be enhanced.

At the same time, the government should leverage the funds obtained from industrial development to promote infrastructure construction. On the one hand, local government can improve the storage and sales efficiency of agricultural products and reduce losses by utilizing agricultural land consolidation and increasing infrastructure such as storage facilities and irrigation systems. On the other hand, by organizing construction land, improving public service infrastructure, and enhancing the convenience and happiness of farmers’ lives, the supporting system, residential system, and human system of rural living environment could be optimized.

(3) Enhancing awareness of vegetation protection and improving ecological restoration work.

The improvement of ecological benefits is a key path to achieving sustainable development goals, especially through enhancing vegetation protection awareness and carrying out ecological restoration work. Faced with the challenges of global environmental change and ecosystem degradation, it is urgent to adopt effective measures to improve vegetation coverage and ecosystem health. Firstly, with the help of ecological restoration, the government and relevant departments should take forward-looking measures to develop and implement large-scale afforestation plans. Based on planting of local tree species and drought tolerant plants, the resilience and self-recovery ability of ecosystems should be enhanced effectively. Secondly, the government and relevant departments should establish monitoring and evaluation mechanisms to ensure the effective implementation of relevant policies. By monitoring the health status and vegetation coverage of the ecosystem regularly, problems should be identified and the corresponding measures should be taked promptly. In addition, scientific research and technological innovation achievements need to be applied to ecological restoration, such as remote sensing technology in monitoring ecological changes and biotechnology in restoring ecosystems. Finally, enterprises and individuals could be motivated to participate in ecological restoration by establishing an ecological compensation mechanism. Enterprises and individuals who have made contributions to ecological restoration could be provided economic compensation or incentives to encourage more social forces to participate in ecological environment protection.

(4) Northern Zhejiang Plain Region (Hangzhou, Jiaxing, Huzhou): Optimizing production space via contiguous smart agriculture to consolidate the material foundation of the human settlement environment.

As a major grain-producing area, the Northern Zhejiang Plain addresses the issue of cultivated land fragmentation through CLC, drawing on Hangzhou’s experience in “digital agriculture” and Jiaxing’s practice of “contiguous construction of permanent basic farmland.” It advances the model of “merging small plots into large ones + cross-village land ownership replacement,” which increases the contiguous scale of cultivated land, raises the annual growth rates of the concentration degree of contiguous cultivated land, land reclamation rate, and grain yield per mu, boosts farmers’ income, and optimizes RHSE. The deployment of “IoT soil moisture monitoring + intelligent drip irrigation” has increased the effective irrigation rate to 95%, while “field service centers” have been established to provide full-process mechanized services, improving the support system. Building on the pilot project in Shuangpu Town of Hangzhou, agricultural cultural experience zones have been planned, raising the proportion of public land to 12%, extending the industrial chain, promoting rural social interaction, and optimizing the social system.

(5) Eastern Zhejiang Coastal Region (Ningbo, Taizhou, Zhoushan): Integrating industrial ecology through land-sea coordination to enhance the multi-dimensional value of the RHSE.

Leveraging its port and coastal resources, the Eastern Zhejiang Coastal Region drives the synergy between industry and ecology via CLC, referencing Ningbo’s practice of “aligning village-level land planning with multiple plans” and Taizhou’s experience in “renovating abandoned mining land.” In Ningbo and Zhoushan, land is reserved in renovated areas for the construction of agricultural product cold chain bases, and the “rice-fish co-culture” model is developed to increase the net grain output value per mu and the added value of aquatic products. In Taizhou and Wenzhou, abandoned salt pans and mine pits have been transformed into “ecological picking gardens + coastal science popularization bases,” which has raised the proportion of public land and farmers’ per capita net income. Through the implementation of “mangrove planting + coastal line restoration,” Zhoushan and Wenzhou have actively restored tidal flat wetlands, improved green vegetation coverage and landscape diversity, and established a coastal ecological compensation mechanism to balance ecology and people’s livelihoods, thereby optimizing the natural and human systems of the RHSE.

(6) Southwestern Zhejiang Mountainous Region (Lishui, Quzhou): Balancing the “Three-Living Spaces” through ecology-prioritized consolidation to improve the ecological and living quality of the RHSE.

Serving as an ecological barrier, the Southwestern Zhejiang Mountainous Region balances ecology and people’s livelihoods through CLC, learning from Lishui’s experience in “terrace restoration” and Quzhou’s practice of “village layout planning.” For sloping farmland with a gradient of less than 25 degrees, “terrace transformation + soil improvement” is implemented, characteristic crops are planted, and large-scale operation is achieved through cooperatives, raising the per-mu income to 3,000 yuan. For abandoned mines, “afforestation + understory planting” is carried out to increase green vegetation coverage. “Hollow villages” are merged to guide the concentrated residence of farmers; idle homesteads are revitalized to build centralized residential areas and “village-level service centers.” Road accessibility has reached the standard of “two-lane roads connecting every village,” and the proportion of public land has been increased. Platforms for “telemedicine + online education” have been built to improve the residential and social systems. Newly added forest land and tea gardens are incorporated into carbon sink trading, with 50% of the proceeds used for village collective public welfare, ensuring the sustainability of the RHSE.

## 6 Conclusion and research prospects

### 6.1 Conclusion

With the research region of Zhejiang Province, China, the influence mechanism of CLC on RHSE from 2000 to 2020 was explored. The main research results are as follows.

(1) The level of CLC and the evolution of RHSE quality in Zhejiang Province. Firstly, from the perspective of provincial level, the development of RHSE and CLC in Zhejiang Province had shown an improved state throughout the entire research stage. From 2000 to 2020. There had been a significant improvement in the level of CLC. The quality of RHSE had significantly improved under the reintegration of production-living-ecological space. Secondly, from the perspective of municipal level, the level of CLC and quality of RHSE had significantly improved in various prefecture level cities in Zhejiang Province from 2000 to 2020. The evaluation results indicated that CLC played a key role in promoting the improvement of RHSE quality and other aspects.
(2) The test results of the influencing mechanism of CLC on RHSE. The single factor detection results of the geographic detector indicated that the average grain yield per mu (x_2_), the proportion of public land (x_5_), the land use elasticity (x_7_), the effective irrigation rate (x_8_), the concentration and connectivity of cultivated land (x_10_), and the land reclamation rate (x_11_) were the most important CLC factors that affected the RHSE. The interaction between the average grain yield per mu (x_2_), effective irrigation rate (x_8_), concentration and connectivity of cultivated land (x_10_), land reclamation rate (x_11_) and other factors was strong relatively, indicating that these indicators had a strong effect on improving RHSE when combined with other factors.
(3) This study explores the influence mechanism of CLC on the quality of RHSE, which is beneficial for promoting the improvement of theories and methods for improving the quality of RHSE. On the other hand, by combining existing experience, differentiated RHSE optimization strategies were constructed. It can provide reference for implementing the rural revitalization strategy and promoting the pilot of CLC.
(4) This study explores the influence mechanism of comprehensive land consolidation on the quality of RHSE. However, due to limitations in time, space, and data, only Zhejiang Province was selected as the case area. Selecting China as the research region to explore how to improve the evaluation index system for RHSE and CLC level requires further in-depth research.

### 6.2 Research prospects

This study still requires further research in the following aspects. Firstly, due to limitations in data availability, only Zhejiang Province, China was selected as the research area. In the future, it is necessary to further expand the research scope to explore the impact of nationwide CLC on RHSE across China. Secondly, this study relies on static data from statistical yearbooks, but recent advancements in the application of dynamic methods—such as remote sensing data integrated with artificial intelligence models—have demonstrated significant improvements (43, 44). Moving forward, the latest dynamic methods will be adopted to measure the impact mechanism of CLC on RHSE.

## Data Availability

The raw data supporting the conclusions of this article will be made available by the authors, without undue reservation.
